# Supplementary material for: Pressure-induced transition from a Mott insulator to a ferromagnetic Weyl metal in La2O3Fe2Se2
Source: Nat Commun. 2023 Apr 20;14:2260. doi: 10.1038/s41467-023-37971-2 (PMC10119149; doi:10.1038/s41467-023-37971-2)
Supplement: Supplementary file 1 — Supplementary information [file 41467_2023_37971_MOESM1_ESM.pdf]

Supplementary Information for

“Pressure-induced transition from a Mott insulator to a ferromagnetic Weyl metal in $La_2O_3Fe_2Se_2$”

Ye Yang[1, †], Fanghang Yu[1, †], Xikai Wen[1], Zhigang Gui[1], Yuqing Zhang[1], Fangyang Zhan[2], Rui Wang[2,*], Jianjun Ying[1, *] and Xianhui Chen[1,3,4, *]

[1]Department of Physics, and CAS Key Laboratory of Strongly-coupled Quantum Matter Physics, University of Science and Technology of China, Hefei, Anhui 230026, China

[2] Department of physics & Center of Quantum Materials and Devices & Chongqing Key Laboratory for Strongly Coupled Physics, Chongqing University, Chongqing 400044, China.

[3]CAS Center for Excellence in Quantum Information and Quantum Physics, Hefei, Anhui 230026, China.

[4]Collaborative Innovation Center of Advanced Microstructures, Nanjing University, Nanjing 210093, China.

[†]These authors contributed equally to this work

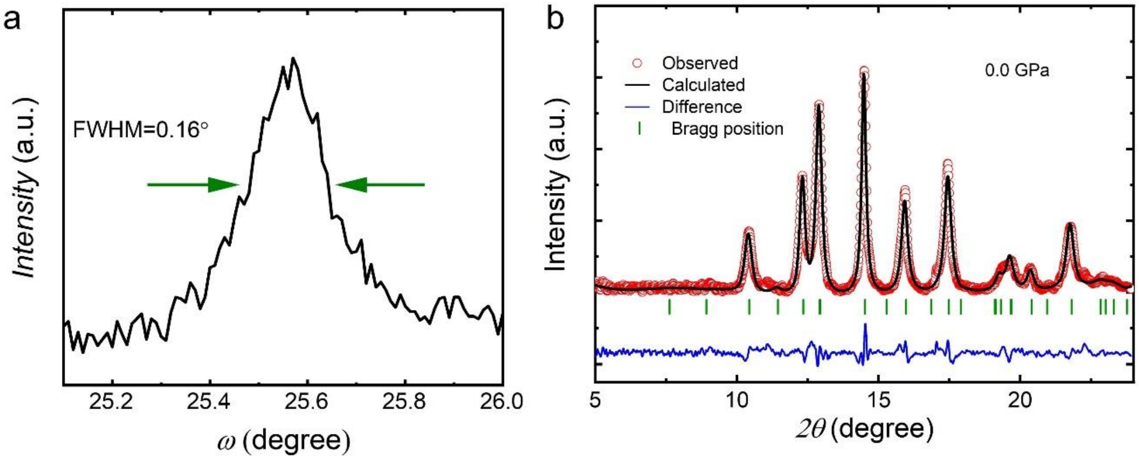

Supplementary Figure 1. a, Rocking curve of the $La_2O_3Fe_2Se_2$ single crystal. The FWHM is 0.16 degree. b, Rietveld fitting of the powder XRD pattern in the diamond anvil cell at 0.0 GPa.

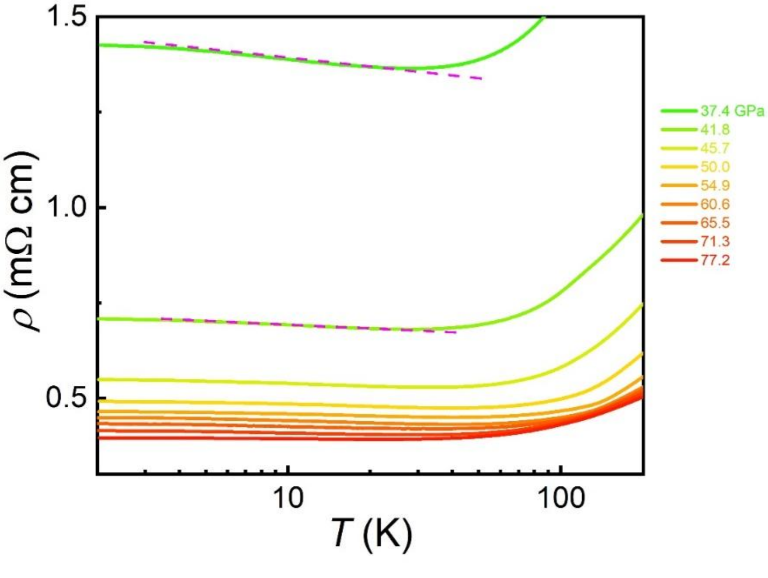

Supplementary Figure 2. Enlarged area of the temperature dependence of resistivity for $La_2O_3Fe_2Se_2$ single crystal. Note that the low-temperature resistivity shows weak upturn when the material becomes metallic. The low-temperature resistivity shows a logarithmic increase and gradually becomes nearly saturated toward low temperatures.

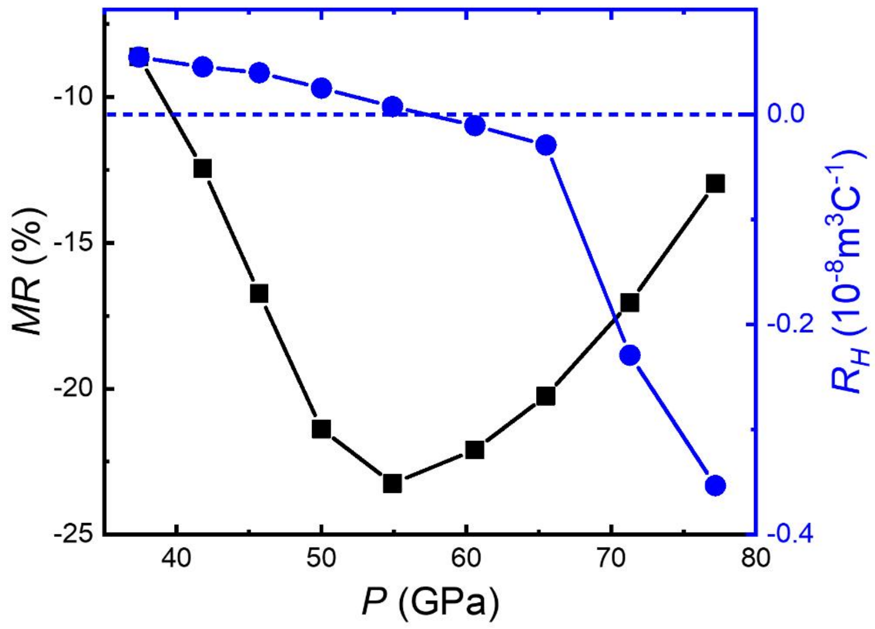

Supplementary Figure 3. Pressure dependence of MR taken at 9 T and 2 K. The blue circles indicate the Hall coefficient measured at 2 K.

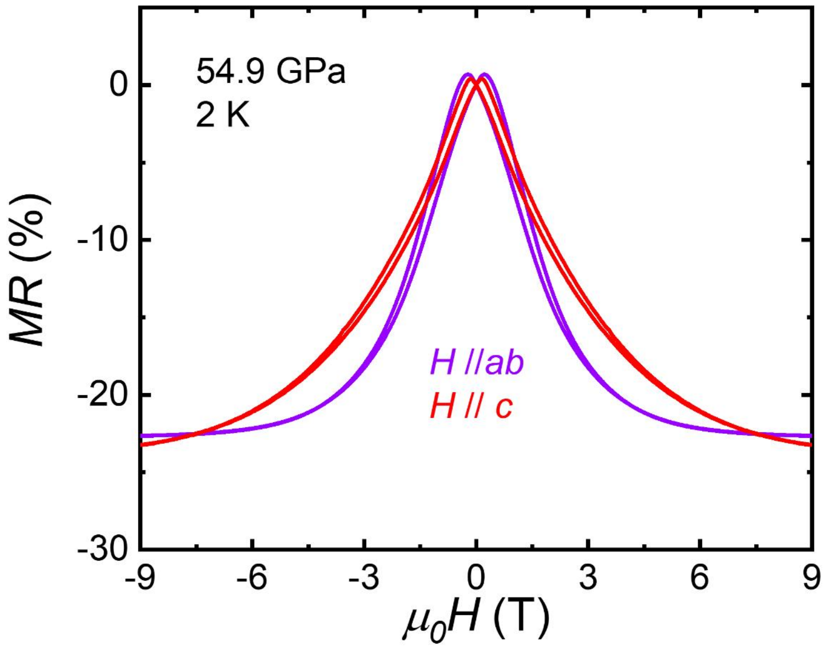

Supplementary Figure 4. The magnetoresistance measured at 2 K and 54.9 GPa with magnetic field applied along ab plane and c axis directions.

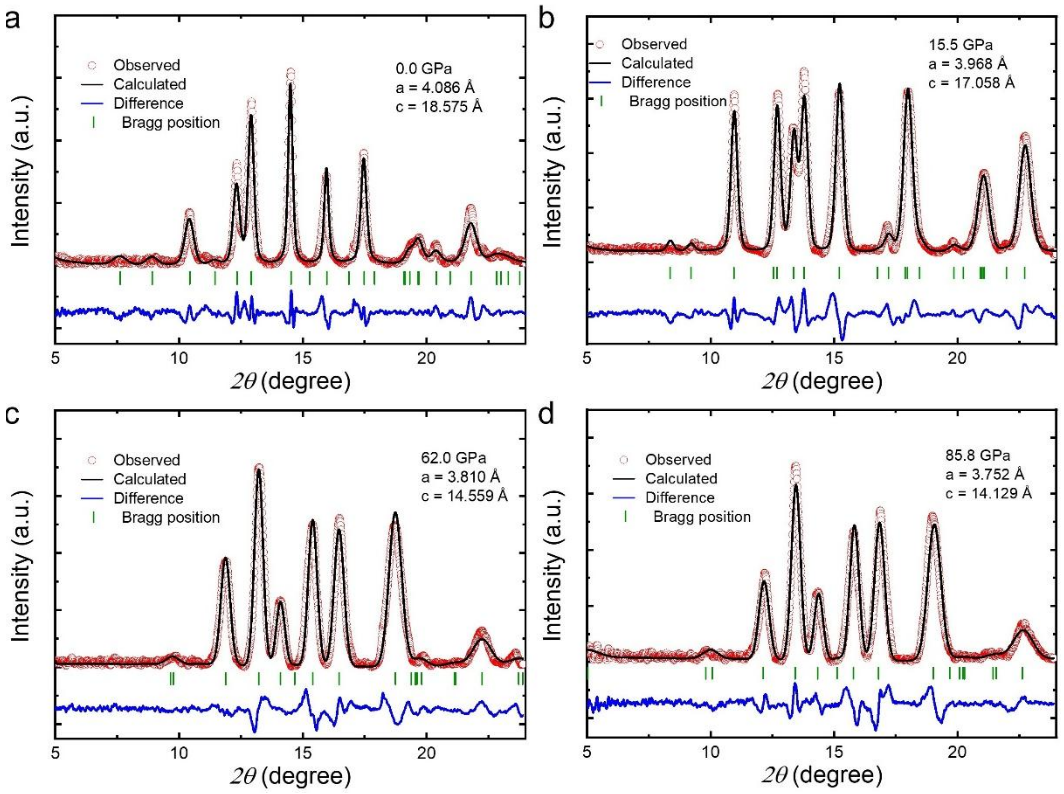

Supplementary Figure 5. Le Bail fitting results for $La_2O_3Fe_2Se_2$ high-pressure XRD patterns with pressure at 0.0 GPa (a), 15.5 GPa (b), 62.0 GPa (c) and 85.8 GPa (d).

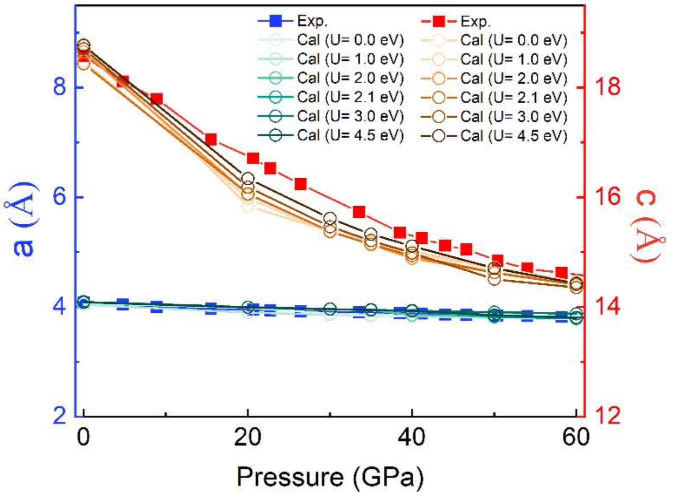

Supplementary Figure 6. The comparison between calculated lattice parameters and experimental values.

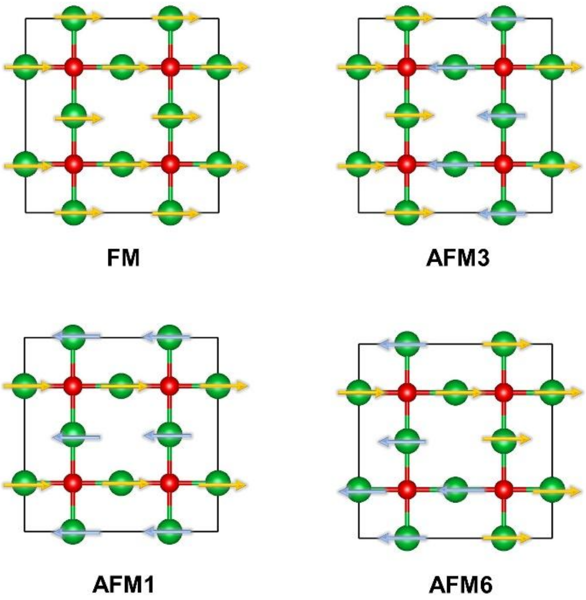

Supplementary Figure 7. The possible magnetic orders of $La_2O_3Fe_2Se_2$ compound.

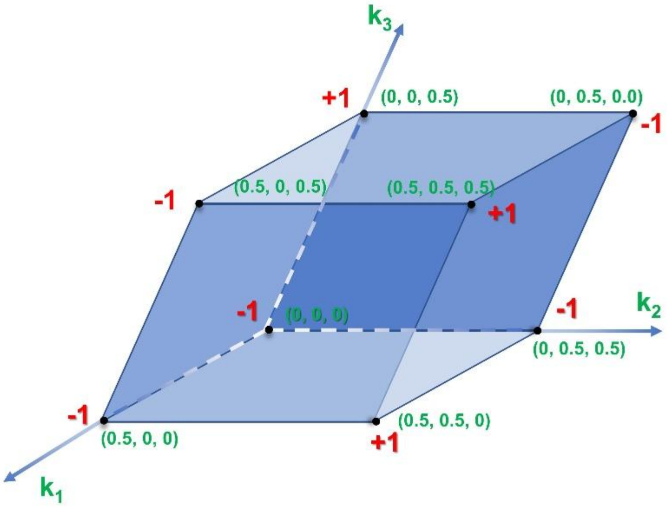

Supplementary Figure 8. Diagram of parity eigenvalues and Chern number for $La_2O_3Fe_2Se_2$ under 48 GPa.

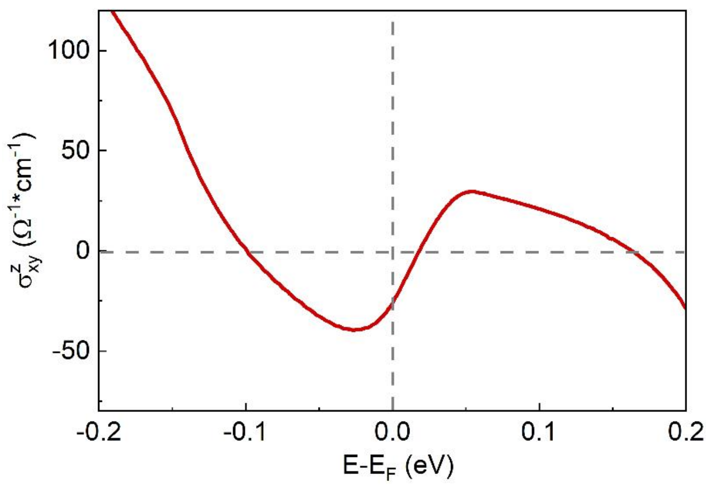

Supplementary Figure 9. The calculated AHC of $La_2O_3Fe_2Se_2$ under 48 GPa.

Supplementary Table 1. The fitting parameters of the $La_2O_3Fe_2Se_2$ sample and comparing with the previous results[1, 2].

	J. Mayer *et al.*	D. Free *et al.*	Our work
a (Å)	4.0788(2)	4.084466(9)	4.080956
c (Å)	18.648(2)	18.59798(7)	18.676563
La *z* (*c*)	0.18445(5)	0.18438(3)	0.166756
Se *z* (*c*)	0.09669(9)	0.09624(3)	0.080705
R_{wp} (%)	4.96	3.95	20.5
χ^2		1.118	2.38

Supplementary Table 2. The chirality, cartesian coordinates and relative energies for Weyl points of $La_2O_3Fe_2Se_2$ under 48 GPa.

chirality	*coordinates* ($Å^{-1}$)	$E-E_F$ *(eV)*
+1	(-0.71989, 0.00006, 0.42874)	-0.01163
+1	(0.50535, -0.81402, 0.00095)	0.03080
+1	(0.50560, 0.81397, 0.00476)	0.03080
+1	(0.73050, 0.00018, -0.00006)	0.00268
+1	(0.81940, -0.51633, -0.00093)	0.03099
+1	(0.82053, 0.51673, 0.00018)	0.03098
-1	(0.71988, 0.00030, -0.42875)	-0.01163
-1	(-0.50520, -0.81404, -0.01326)	0.03080
-1	(-0.50543, 0.81403, 0.01104)	0.03080
-1	(-0.73047, 0.00021, 0.00002)	0.00268
-1	(-0.81872, -0.51616, -0.00024)	0.03099
-1	(-0.82064, 0.51670, -0.00191)	0.03098

Supplementary References

1. Mayer J. M., Schneemeyer L. F., Siegrist T., Waszczak J. V., Van Dover B. New Layered Iron-Lanthanum-Oxide-Sulfide and -Selenide Phases: $Fe_2La_2O_3E_2$(E= S,Se). *Angew. Chem. Int. Ed. Engl.* **31**, 1645-1647 (1992).

2. Free D. G., Evans J. S. O. Low-temperature nuclear and magnetic structures of $La_2O_2Fe_2OSe_2$ from x-ray and neutron diffraction measurements. *Phys. Rev. B* **81**, 214433 (2010).
